# Compression Helps Deep Learning in Image Classification

**DOI:** 10.3390/e23070881

**Published:** 2021-07-10

**Authors:** En-Hui Yang, Hossam Amer, Yanbing Jiang

**Affiliations:** Department of Electrical and Computer Engineering, University of Waterloo, Waterloo, ON N2L 3G1, Canada; ehyang@uwaterloo.ca (E.-H.Y.); yanbing.jiang@uwaterloo.ca (Y.J.)

**Keywords:** image compression, deep learning, inception network, residual network, JPEG

## Abstract

The impact of JPEG compression on deep learning (DL) in image classification is revisited. Given an underlying deep neural network (DNN) pre-trained with pristine ImageNet images, it is demonstrated that, if, for any original image, one can select, among its many JPEG compressed versions including its original version, a suitable version as an input to the underlying DNN, then the classification accuracy of the underlying DNN can be improved significantly while the size in bits of the selected input is, on average, reduced dramatically in comparison with the original image. This is in contrast to the conventional understanding that JPEG compression generally degrades the classification accuracy of DL. Specifically, for each original image, consider its 10 JPEG compressed versions with their quality factor (QF) values from {100,90,80,70,60,50,40,30,20,10}. Under the assumption that the ground truth label of the original image is known at the time of selecting an input, but unknown to the underlying DNN, we present a selector called Highest Rank Selector (HRS). It is shown that HRS is optimal in the sense of achieving the highest Top *k* accuracy on any set of images for any *k* among all possible selectors. When the underlying DNN is Inception V3 or ResNet-50 V2, HRS improves, on average, the Top 1 classification accuracy and Top 5 classification accuracy on the whole ImageNet validation dataset by 5.6% and 1.9%, respectively, while reducing the input size in bits dramatically—the compression ratio (CR) between the size of the original images and the size of the selected input images by HRS is 8 for the whole ImageNet validation dataset. When the ground truth label of the original image is unknown at the time of selection, we further propose a new convolutional neural network (CNN) topology which is based on the underlying DNN and takes the original image and its 10 JPEG compressed versions as 11 parallel inputs. It is demonstrated that the proposed new CNN topology, even when partially trained, can consistently improve the Top 1 accuracy of Inception V3 and ResNet-50 V2 by approximately 0.4% and the Top 5 accuracy of Inception V3 and ResNet-50 V2 by 0.32% and 0.2%, respectively. Other selectors without the knowledge of the ground truth label of the original image are also presented. They maintain the Top 1 accuracy, the Top 5 accuracy, or the Top 1 and Top 5 accuracy of the underlying DNN, while achieving CRs of 8.8, 3.3, and 3.1, respectively.

## 1. Introduction

Deep learning (DL) is becoming increasingly ubiquitous in the task of image classification due to its ability to extract desired features from raw data [1,2,3,4,5,6,7,8,9,10]. DL is created through cascading non-linear layers that progressively produce multi-layers of representations with increasing levels of abstraction, starting from the raw input data and ending with the predicted output label [5,7,11,12,13,14,15,16]. These multi-layers of representations are features not designed by human engineers with considerable domain expertise, but they are learned from the raw data through a backpropagation learning algorithm.

In image classification, the raw data fed into a DL machine are the pixel values of an image to be classified. Note that the meaning of raw data in the context of DL here is with respect to subsequently extracted features but not in the context of compression. In the whole pipeline of data acquisition, data encoding (i.e., compression), data transmission, and data processing/utilization, the raw data fed into a DL machine are not “raw”; instead, they are generally compressed in a lossy manner. Since lossy compression is about the trade-off between compression ratio (CR) and compression quality, many versions of compressed raw data in the context of DL can be produced with each version having a different compression ratio and compression quality. This in turn brings forth the following interesting question to DL:

**Question** **1** Which version of compressed raw data is good for DL and its related applications?

In practice, images are often compressed by JPEG encoders [17,18,19]. For most practical applications with JPEG, both the CR and compression quality of a JPEG image are controlled by a parameter called the quality factor (QF); the higher is the QF, the lower is the CR and the better is the compression quality. With the maximum value of QF at 100, the majority of JPEG images in the ImageNet Large Scale Visual Recognition Challenge (ILSVRC) 2012 dataset [20,21] have high QF values ranging from 91 to 100, implying that they all have high compression quality.

**Figure 1:**
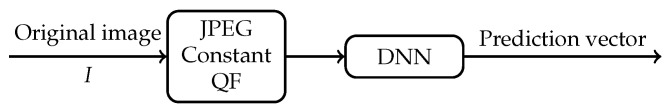
A DNN with a JPEG compressed version of an image as an input, where QF is a constant.

In the literature, Question 1 is investigated to some extent on the basis of constant QFs which are the same for all images in a whole set of JPEG images [22,23,24,25,26,27], as shown in Figure 1. Specifically, four deep neural network (DNN) models were tested by Dodge et al. [23] on a subset of the validation set of the ILSVRC 2012 dataset [20]. To evaluate the impact of compression on the classification performance of these four DNN models, all images in the subset were further compressed by JPEG with the same constant QF. These compressed images with the constant QF were then fed into each of these four DNN models. Both the Top 1 classification accuracy and the Top 5 classification accuracy were recorded. As the value of the constant QF decreases, the curves of the Top 1 classification accuracy vs. QF and the Top 5 classification accuracy vs. QF were plotted by Dodge et al. [23] in the QF range from 20 to 2. It was shown by Dodge et al. [23] that both the Top 1 classification accuracy and the Top 5 classification accuracy of each of the four DNN models decay as the value of the constant QF decreases. This phenomenon of negative impact of compression on the classification performance of DNN models was also reported by Liu et al. [27].

To alleviate the negative impact of JPEG compression on the classification performance of DNN models to some extent, several methods were proposed in the literature, including data augmentation, stability training, and due-channel training with preprocessing [24,27,28,29,30,31]. For example, stability training was proposed by Zheng et al. [24], where, during the training stage of a DNN model, both the original image and its distorted version are fed into the model, and training is performed to minimize a modified cost function which takes stability into consideration. Although these methods improve the classification robustness of DNN models against JPEG compression and other types of distortion, there is still a significant degradation (as high as 10%) in the classification accuracy when these newly trained DNN models are applied to low-quality JPEG compressed images. Based on these findings, it is generally believed that compression, especially JPEG compression, would hurt the classification accuracy of deep learning in image classification.

**Figure 2:**
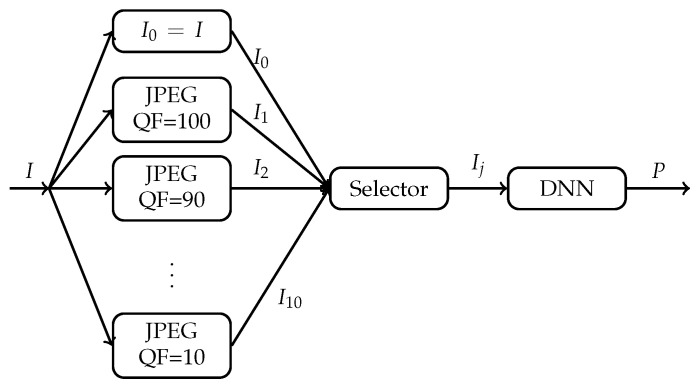
Selection of a compressed version of an image as an input to a given DNN, where *P* is the prediction vector of the DNN in response to the chosen $I_{j}$.

In this paper, we investigate Question 1 in the context of JPEG compression from a different perspective. Instead of using a constant QF in JPEG compression for all images in the ILSVRC 2012 dataset, we would allow each image to be compressed first with a possibly different QF and then fed into a DNN. Specifically, let QF take values from {100,90,80,70,60,50,40,30,20,10} (This set of QF values is simply used as an example. The idea of this paper, however, can be applied to any set of QF values. In addition, QF=10 is regarded in this example as the lowest compression quality acceptable to humans.) We associate each original image in the ILSVRC 2012 dataset with its 10 compressed versions, each compressed version corresponding to a different QF from {100,90,80,70,60,50,40,30,20,10}. For each image, there are now 11 different versions: 1 original version and 10 compressed versions. As shown in Figure 2, for each original image *I*, we now have freedom to select one version $I_{j}$ out of its 11 versions $I_{i}$, i=0,1,⋯,10, to be fed into the DNN. Is there any selector that can select, for each original image *I*, a suitable version $I_{j}$ to be fed into the DNN so that both the Top 1 classification accuracy and Top 5 classification accuracy of the DNN can be improved significantly while the size (in bits) of the input image to the DNN can be reduced dramatically in general?

One of our purposes in this paper is to settle the above question. We show that the answer to the above question turns out to be positive. Therefore, in contrast to the conventional understanding, compression, if used in the right manner, actually improves the classification accuracy of a DNN significantly while also reducing dramatically the number of bits needed to be fed into the DNN. Specifically, a DNN pre-trained with pristine ImageNet images is fixed. That is, the DNN is trained with the original images in the training set of the ILSVRC 2012 dataset. Suppose that the ground truth label of each original image *I* is known to the selector in Figure 2, but it is unknown to the DNN. Under this assumption, we propose a selector called Highest Rank Selector (HRS). For each original image *I*, HRS works as follows. Examine the prediction vector $P_{i}$ of the DNN in response to each version $I_{i}$; determine the rank of the ground truth label in the sorted $P_{i}$, where labels in $P_{i}$ are sorted according to their probabilities in descending order with rank 1 being the highest ranking; and then select the compressed version $I_{j}$ as the desired input to the DNN if the rank of the ground truth label in the sorted $P_{j}$ is the highest among all sorted $P_{i}$, where, in the case of tie, HRS selects the compressed version with the lowest QF. It can be shown that, among all selectors one could possibly design, HRS achieves the highest Top 1 and Top 5 classification accuracy and hence is optimal. When applied to Inception V3 and ResNet-50 V2 architectures pre-trained with pristine ImageNet images [32,33], HRS improves, on average, the Top 1 classification accuracy by 5.6% and the Top 5 classification accuracy by 1.9% on the whole ImageNet validation set. In addition, compared with the original image, the compressed version selected by HRS also achieves, on average, the CR of 8.

When the ground truth label of each input image is unknown to the selector in Figure 2 as well, HRS is not applicable. To demonstrate that compression still improves the classification accuracy of DL in this case, we propose a new convolutional neural network (CNN) topology based on a given DNN. Consider the main architecture of the DNN without its last fully connected layer. As shown in Figure 12, the new CNN topology based on the given DNN consists of 11 parallel main architectures of the DNN followed by the last fully connected layer at which the logit blocks from the 11 parallel main architectures are concatenated. The original image and its 10 compressed versions are inputs to the 11 parallel main architectures of the given DNN, respectively. These 11 parallel identical main architectures are first pre-trained with the original images in the training set of the ILSVRC 2012 dataset. The last a few layers of the new CNN topology are then re-trained. Experimental results show that, when compared with the given underlying DNN, the new CNN topology improves the Top 1 classification accuracy and the Top 5 classification accuracy by 0.4% and 0.3%, respectively, when the given underlying DNN is Inception V3 and by 0.4% and 0.2%, respectively, when the given underlying DNN is ResNet-50 V2.

In another direction, when the ground truth label of each input image is unknown to the selector in Figure 2, we also propose a selector which can maintain the same the Top 1 classification accuracy and Top 5 classification accuracy as those of the given DNN with the original image as its input. When applied to Inception V3 and ResNet-50 V2, the compressed version selected by the proposed selector achieves, on average, the CR of 3.1 in comparison with the original image.

The remainder of the paper is organized as follows. Section 2 provides a case study that motivates the research work in this paper. In Section 3, we describe HRS in detail, demonstrate its optimality, and analyze why it improves classification accuracy significantly while achieving dramatic reduction in bits. Section 4 and Section 5 are devoted to the case where the ground truth label of each input image is unknown to the selector in Figure 2, with Section 4 focusing on the new CNN topology. Section 5 is devoted to the proposed selector maintaining classification accuracy while achieving significant reduction in bits. Finally, Section 6 concludes the paper.

## 2. Motivation: Case Study

This section motivates our approach to Question 1 as illustrated in Figure 2. Let us first reproduce results which lead people to the conventional understanding that JPEG compression generally degrades classification performance of DNNs.

**Figure 3:**
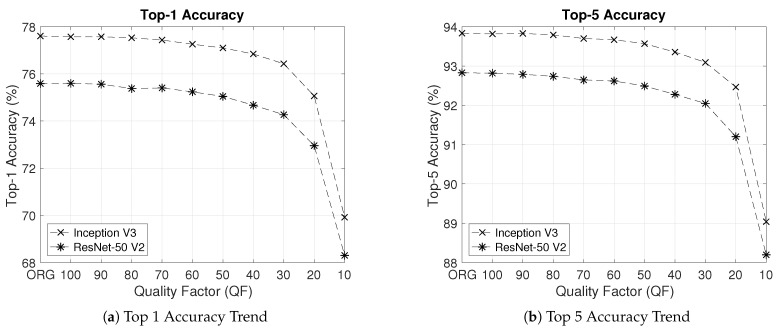
Top 1 accuracy and Top 5 accuracy degradation phenomenon for Inception V3 and ResNet-50 V2 in the case of the “one QF vs. all images” approach.

The conventional understanding is based on the approach shown in Figure 1, where a constant QF is used to compress all images in a whole set of images. This approach can be dubbed as “one QF vs. all images”. For Inception V3 and ResNet-50 V2 pre-trained with the original images in the training set of the ILSVRC 2012 dataset, Figure 3 shows their respective curves of the Top 1 classification accuracy and Top 5 classification accuracy on the whole ImageNet validation dataset vs. the constant QF as the value of the constant QF in Figure 1 decreases from 100 to 10 with a step size of 10. From the results in Figure 3, it is clear that classification performance deteriorates as the value of the constant QF decreases, hereby reconfirming the conventional understanding.

**Figure 4:**
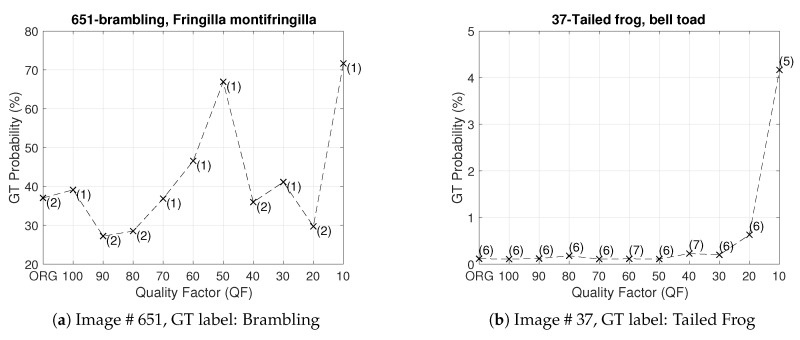
The perspective of one image vs. all QFs—the ranks and probabilities of the GT label of an image across different QFs: (**a**) Image # 651; and (**b**) Image # 37.

**Figure 5:**
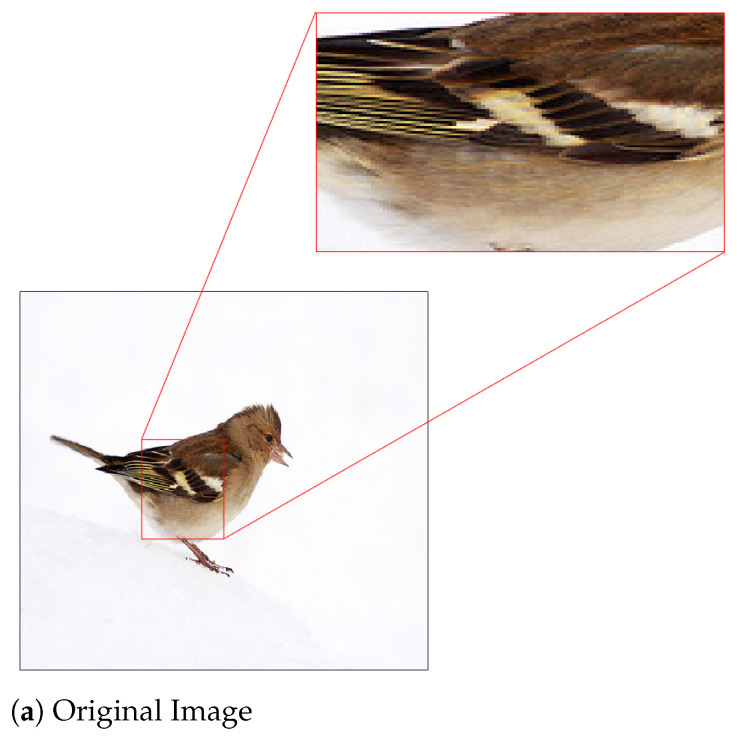

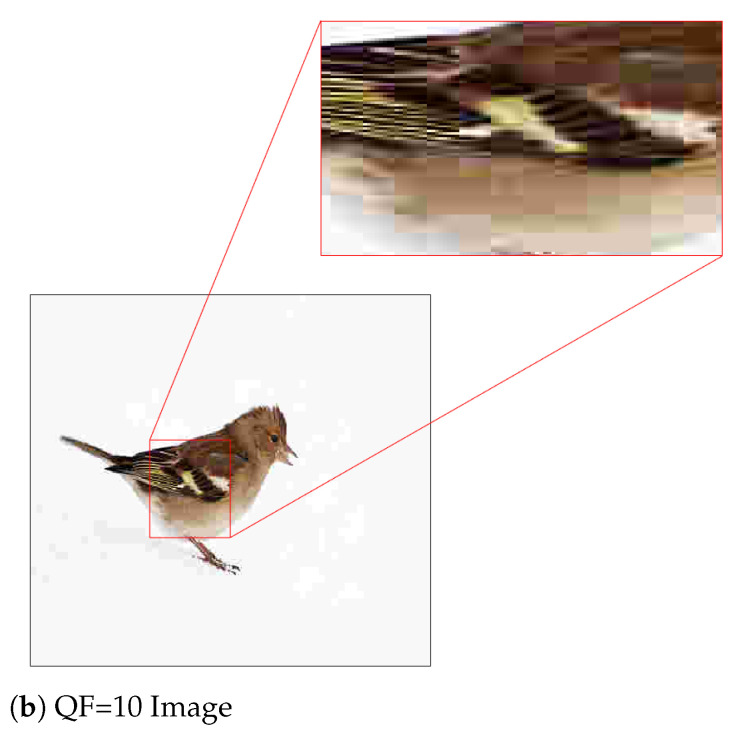
Image #651 from ImageNet validation set with its GT GT label “Brambling”: (**a**) the original image for which the GT label ranks second with probability 37%; and (**b**) the JPEG compressed image with QF = 10 for which the GT label ranks first with probability 72%. Best viewed in electronic format.

Note that the concept of classification accuracy is a group notion with respect to a whole set of images. If, however, we focus on a particular image and examine the impact of JPEG compression with different QFs on the predicted vector of the underlying DNN—such a perspective is dubbed as “one image vs. all QFs”—then the rank and probability of the ground truth (GT) label of the image in the predicted vector do not necessarily go down as the value of QF decreases. This is indeed confirmed by Figure 4. With Inception V3 pre-trained with ImageNet pristine images as the underlying DNN, Figure 4 shows the ranks and probabilities of the GT labels of Images #651 and #37 in the ImageNet validation set as the value of QF decreases. In this Figure, it is clear that, for a given image, a JPEG compressed version with a lower QF could yield a higher rank of the GT label and a larger probability of the GT label in comparison with the original image. For example, for Image #651 shown in Figure 5, when the original image is fed into the underlying DNN, the GT label ranks second with probability 37% in the corresponding predicted vector. On the other hand, when its JPEG compressed version with QF=10 shown in Figure 5 is fed into the underlying DNN, the GT label ranks first with probability 72% in the corresponding predicted vector; both the rank and probability of the GT label are improved. The same phenomenon is observed for Image #37 and in the case of the ResNet-50 V2 architecture as well.

**Figure 6:**
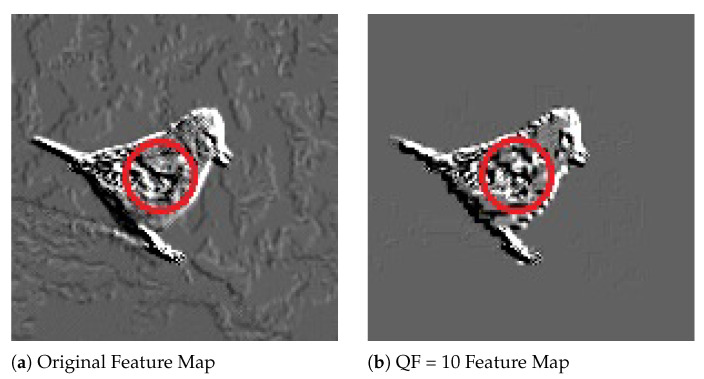
Feature Maps extracted from the original Image #651 and its JPEG compressed version with QF = 10 by Layer 1 of Inception V3. Best viewed in electronic format.

To shed light on why, for a particular image, both the rank and probability of its GT label resulting from a JPEG compressed version with a low QF could be higher than those resulting from the original image, Figure 6 shows a pair of corresponding feature maps extracted from the original Image #651 and its JPEG compressed version with QF=10, respectively, by Layer 1 of Inception V3. In Figure 6, the feature map extracted from the JPEG compressed image with QF=10 is a lot of cleaner and has much better contrast between the foreground and background than the one extracted from the original image. This is likely due to the unequal quantization performed by JPEG on different discrete cosine transform (DCT) coefficients, which is non-linear and reduces more energy in the background than the foreground. This, combined with the subsequent rectified linear unit (ReLU) function in Inception V3, essentially wipes out the background information.

The above case study suggests that, if for any image, one can select, among its many compressed versions including its original version, a suitable version as an input to the underlying DNN, then the classification accuracy of the underlying DNN could be improved. In addition, if a highly compressed version is selected most of the time, then the size in bits of the input is also reduced dramatically in comparison with the original image. The question, of course, is how to select such a compressed version, which is addressed in the next section when the GT label of the image is known to the selector.

## 3. Highest Rank Selector

With reference to Figure 2, in this section, we assume that the GT label of each original image is known to the selector, but unknown to the underlying DNN. We present Highest Rank Selector (HRS) and demonstrate its optimality in the sense of achieving the highest classification accuracy for a given underlying DNN among all possible selectors. We also provide empirical analysis on the performance of HRS in terms of classification accuracy improvement and compression ratio for Inception V3 and ResNet-50 V2 pre-trained with the original images in the training set of the ILSVRC 2012 dataset.

### 3.1. HRS and Its Optimality

An underlying DNN is fixed. As illustrated in Figure 2, each original image *I* is now associated with 11 JPEG compressed images (including the original image itself) $I_{i}$, i=0,1,⋯,10. Let $P_{i}$ denote the prediction vector of the underlying DNN in response to the input $I_{i}$. HRS now works as follows:

**Step** **1** For each 0≤i≤10, determine the rank $r_{i}$ of the GT label of *I* in the sorted $P_{i}$, where labels in $P_{i}$ are sorted according to their probabilities in descending order with rank 1 being the highest ranking.
**Step** **2** Select $I_{j}$ as an input to the underlying DNN if and only if
  (1) $$r_{j}=\min\left\{r_{i}:0\leq i\leq 10\right\}$$
  where, whenever there are multiple *i*s achieving the above minimum, *j* is selected to be the largest among those *i*s.

**Example** **1.** *Let the underlying DNN be Inception V3 pre-trained with the original images in the training set of the ILSVRC 2012 dataset. For Image # 651, in view of Figure 4, HRS selects $I_{10}$, the JPEG compressed image with QF = 10 as an input to the underlying DNN. For Image # 37, the same is true as well since, for Image # 37, $r_{10}$ is the smallest, as shown again in Figure 4.*

For any selector *S*, let $P_{S}\left(I\right)$ denote the prediction vector at the output of the system shown in Figure 2 with *S* as the selector in response to *I*. Let $r_{S}\left(I\right)$ be the rank of the GT label of *I* in the sorted $P_{S}\left(I\right)$. For any set of images A, let $A_{S}\left(k\right)$ denote the Top *k* classification accuracy of the system shown in Figure 2 with *S* as the selector on the image set A. For convenience, $A_{S}\left(k\right)$ is referred to as the Top *k* accuracy of the selector *S* on the image set A as well in the rest of the paper. The following theorem implies that HRS achieves the highest Top *k* accuracy on any image set A among all possible selectors, and hence is optimal.

**Theorem** **1.** *For any image set A, any selector S, and any k, the following holds*

(2) $$A_{HRS}\left(k\right)\geq A_{S}\left(k\right)$$

**Proof** **of Theorem 1.** For any image I∈A, it follows from (1) that

$$r_{HRS}\left(I\right)\leq r_{S}\left(I\right)$$

which further implies

$$\left\{I\in A:r_{HRS}\left(I\right)\leq k\right\}⊇\left\{I\in A:r_{S}\left(I\right)\leq k\right\}$$

Therefore,

$$\begin{array}{ccc} A_{HRS}\left(k\right) & = & \frac{\left|\{I\in A:r_{HRS}\left(I\right)\leq k\}\right|}{\left|A\right|}\\ & \geq & \frac{\left|\{I\in A:r_{S}\left(I\right)\leq k\}\right|}{\left|A\right|}\\ & = & A_{S}\left(k\right) \end{array}$$

where, for any set *C*, |C| denotes the cardinality of *C*. This completes the proof of Theorem 1. □

Before we conclude this subsection, let us mention an application scenario where the GT label of the image *I* is indeed known to the selector in Figure 2, but unknown to the underlying DNN. Consider, for example, a party gathering with a lot of participants and high security requirements. Before the party gathering, each invited participant is requested to provide his/her high quality photo along with his/her identification (ID) information to the party organizer. Upon receiving the photo and ID information of an invited participant, the organizer later on issues to the invited participant a formal invitation letter with the photo (possibly further compressed by JPEG) embedded in a chip. At the time of party gathering, each invited participant will go through security by presenting the invitation letter to an underlying DNN, which in turn reads the photo inside the chip of the letter to determine the ID of the invited participant. In this case, the organizer can act as a selector with the knowledge of the ID of each invited participant (i.e., the GT label of the original high quality photo corresponding to the invited participant), which is unknown to the underlying DNN; the photo put inside the chip is the JPEG compressed version selected by the selector. Both the classification accuracy of the selector and the size in bits of the selected JPEG compressed image inside the chip of each letter are important.

### 3.2. Empirical Results and Analysis

Table 1 tabulates the Top 1 and Top 5 accuracy results of HRS on the whole ImageNet validation dataset for Inception V3 and ResNet-50 V2 pre-trained with the original images in the training set of the ILSVRC 2012 dataset, respectively. As shown in this table, the average accuracy improvement for Inception V3 and ResNet-50 V2 is 5.6% in terms of Top 1 accuracy and 1.9% in terms of Top 5 accuracy.

**Figure 7:**
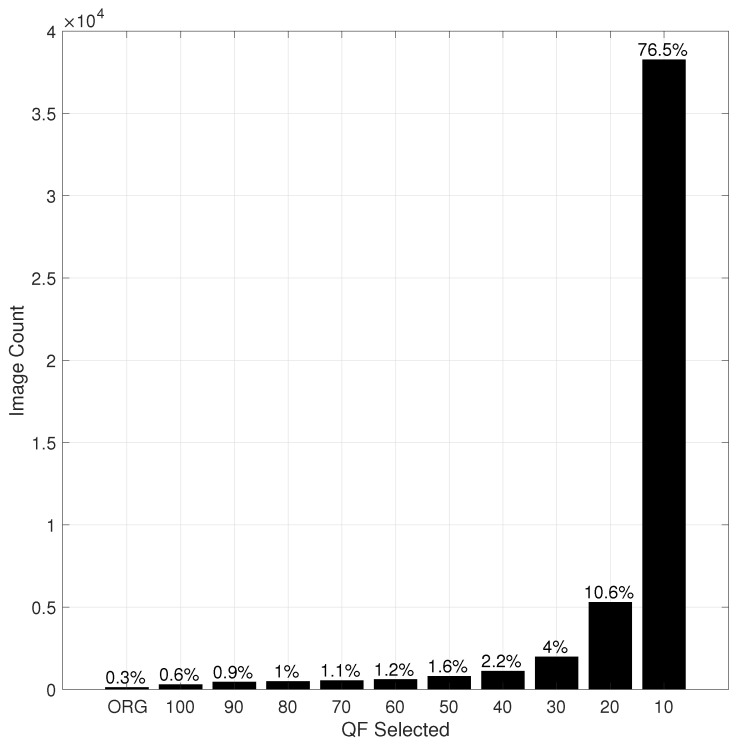
The histogram of QF values selected by HRS for Inception V3.

**Figure 8:**
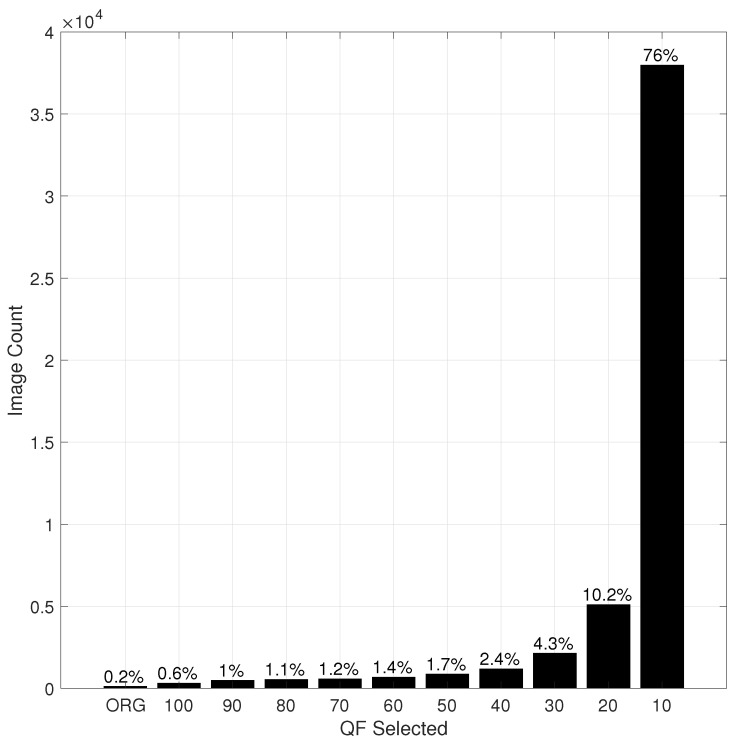
The histogram of QF values selected by HRS for ResNet-50 V2.

Figure 7 and Figure 8 show the histograms of the QF values selected by HRS for Inception V3 and ResNet-50 V2, respectively. It is observed in Figure 7 and Figure 8 that, in both cases, a JPEG compressed version with a lower QF is selected by HRS more often than its counterpart with a higher QF. For the set of QF values {100, 90, 80, 70, 60, 50, 40, 30, 20. 10}, the lowest QF value (i.e., QF10) was the most selected QF. However, this is not necessarily true in general, when a large set containing many small QF values is used for selection. This phenomenon in turn translates into a dramatic reduction in the size (in bits) of the selected input to the underlying DNN in comparison with the original image, as shown in Table 2, where the default size is the total size in Gigabytes (GB) of all original images in the ImageNet validation dataset, and the HRS size is the total size of all selected images by HRS. The compression ratio achieved by HRS is, on average, 8.

Now, take A to be the whole ImageNet validation dataset. Let *D* be the default selector which always selects the original image *I* as an input to the underlying DNN. Note that, for any $I\in \left\{I\in A:r_{HRS}\left(I\right)\leq k\right\}-\left\{I\in A:r_{D}\left(I\right)\leq k\right\}$, HRS improves the rank of the GT label of *I* from being below Top *k* to Top *k*. To have a better understanding on HRS, let us examine, through examples, feature maps extracted by some layers of the underlying DNN from the original image *I* and the compressed image selected by HRS, respectively, for $I\in \left\{I\in A:r_{HRS}\left(I\right)\leq k\right\}-\left\{I\in A:r_{D}\left(I\right)\leq k\right\}$. To be specific, take the underlying DNN to be Inception V3 pre-trained with the original images in the training set of the ILSVRC 2012 dataset.

**Figure 9:**
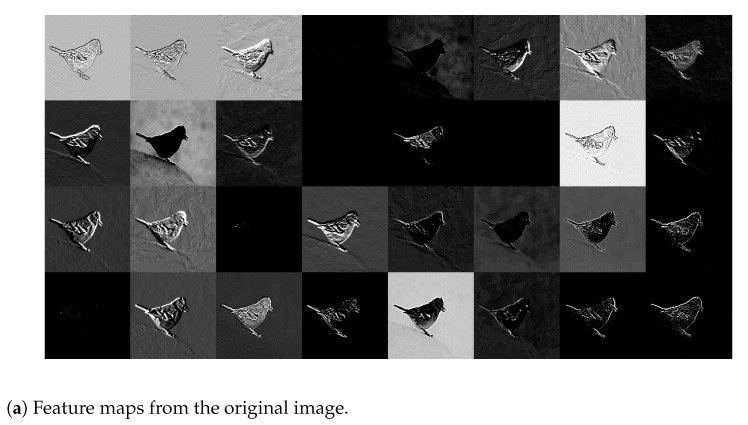

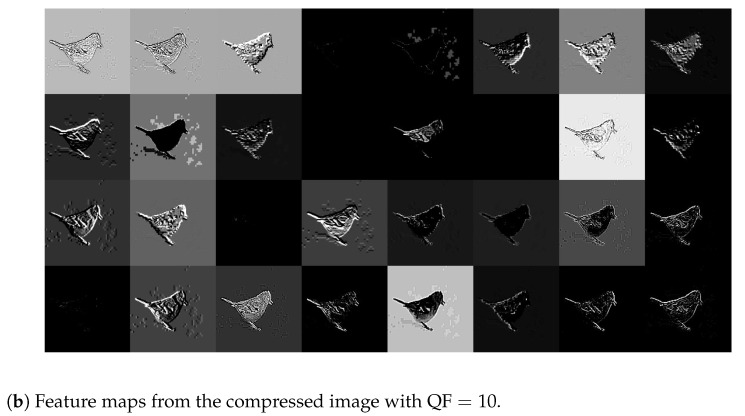
Feature maps extracted by Layer 2 of Inception V3 from the original Image#651 with GT label “Brambling” from the ImageNet validation dataset and its JPEG compressed version with QF = 10. Best viewed in electronic format.

**Example** **2.** *Let I be Image # 651. In view of Figure 4, HRS selects $I_{10}$ and $r_{HRS}\left(I\right)=1$, while $r_{D}\left(I\right)=2$. Therefore, $I\in \left\{I\in A:r_{HRS}\left(I\right)\leq 1\right\}-\left\{I\in A:r_{D}\left(I\right)\leq 1\right\}$. Figure 9 shows feature maps extracted by Layer 2 of Inception V3 from the original Image #651 and the compressed image $I_{10}$ selected by HRS, respectively. In this figure, it can be observed that feature maps extracted from the compressed image are generally a lot of cleaner and have much better contrast between the foreground information, i.e., the bird, and the background information than their counterparts from the original image. For any particular pair of feature maps extracted from the original image and the compressed image $I_{10}$, respectively, the bird is either visible or invisible in both of them. Whenever the bird is visible, the background of the feature map extracted from the compressed image is simple or less wiped out, whereas the background of the feature map extracted from the original image is complicated and still contains significant energy most of the time. These differences will be propagated to subsequent layers of Inception V3. It is these difference along with the clearer texture on the body of the bird in the compressed image (see Figure 5) that makes the underlying DNN to distinguish a Brambling bird from a Junco snowbird that was originally ranked first in the probability vector produced by the original image.*

**Figure 10:**
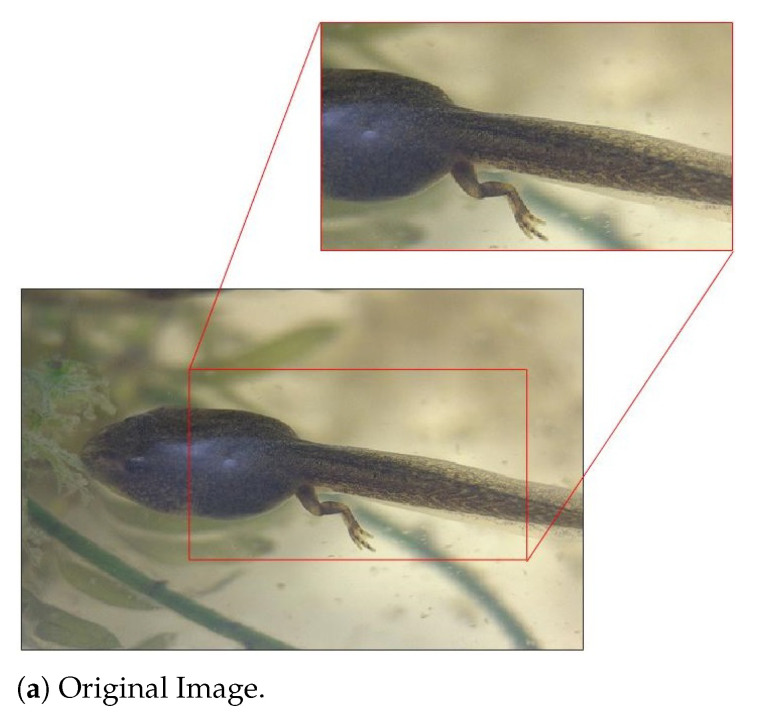

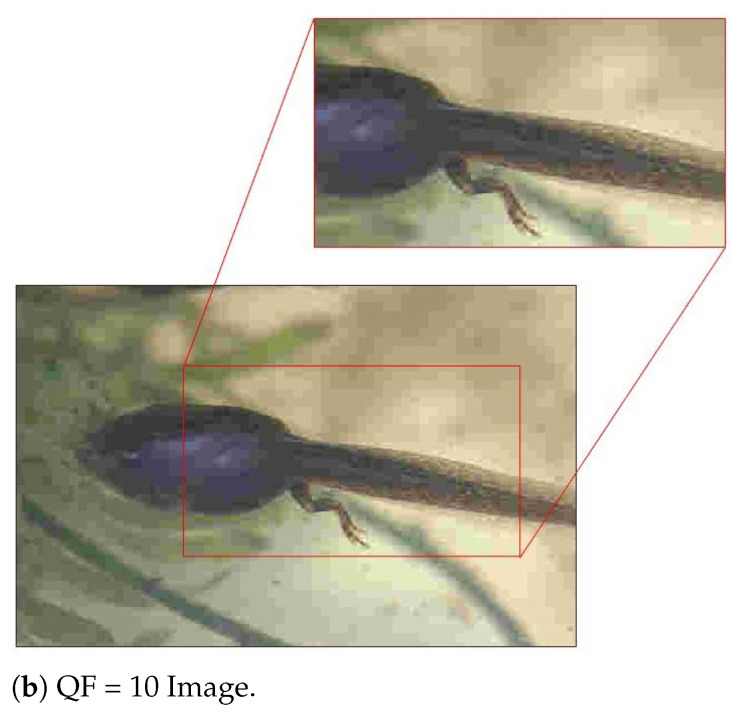
Image #37 from the ImageNet Validation dataset with its GT label “Tailed frog”: (**a**) the original image; and (**b**) the JPEG compressed version with QF = 10. Best viewed in electronic format.

**Figure 11:**
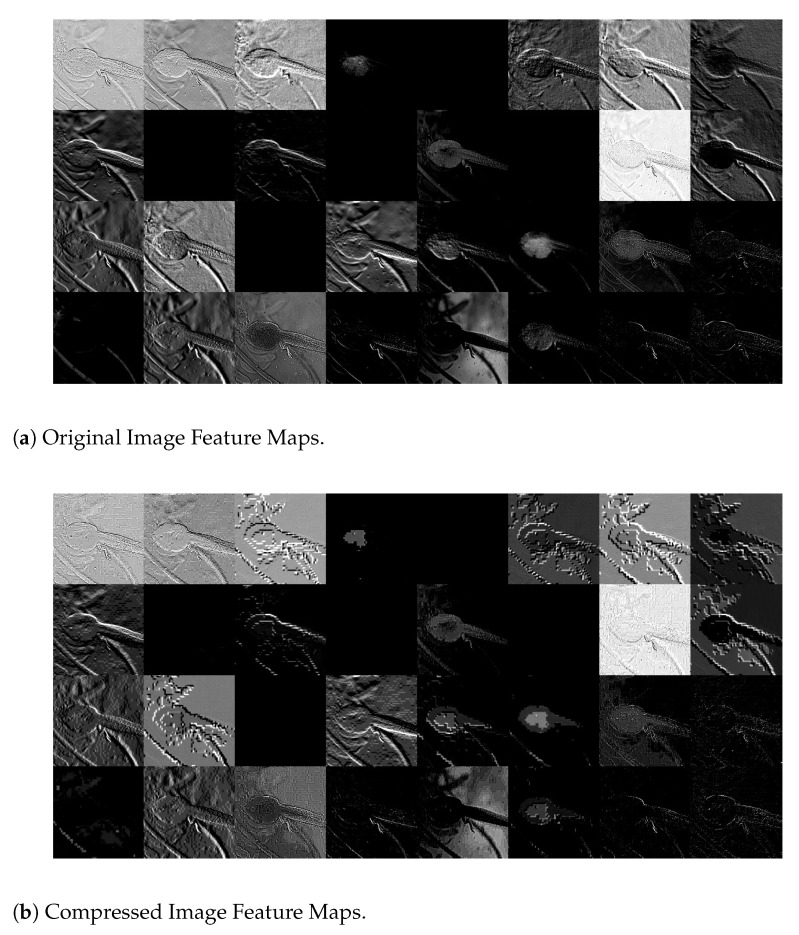
Feature maps extracted by Layer 2 of Inception V3 from the original Image#37 with GT label “Tailed Frog” from the ImageNet validation dataset and its JPEG compressed version with QF=10. Best viewed in electronic format.

**Example** **3.** *Let I be Image # 37. In view of Figure 4, HRS selects $I_{10}$ and $r_{HRS}\left(I\right)=5$, while $r_{D}\left(I\right)=6$. Therefore, $I\in \left\{I\in A:r_{HRS}\left(I\right)\leq 5\right\}-\left\{I\in A:r_{D}\left(I\right)\leq 5\right\}$. Figure 10 shows the original image I and its JPEG compressed version with QF=10. Their respective feature maps extracted by Layer 2 of Inception V3 are illustrated in Figure 11. In these figures, the same understanding as explained in Example 2 can be confirmed. In feature maps extracted from the JPEG compressed version with QF=10, the contrast is improved and interference information such as tiny spots on the frog’s body is removed. In addition, key features for the frog, such as the outlier of its body, are retained. The tiny spots in the frog’s body make Image #37 confused with a spotted salamander, which was ranked fifth with the original input image. Again, all of these differences will be propagated to subsequent layers of Inception V3.*

To summarize, we show above that, if a JPEG compressed version of an image is selected on an individual image base as an input to an underlying DNN and the GT label of the original image is known to the selector (but unknown to the underlying DNN), the classification performance of the underlying DNN can be improved significantly while the size in bits of the input image can be reduced dramatically. Therefore, in contrast to conventional understanding, JPEG compression indeed helps DL in image classification. In the next section, we demonstrate that this is also true even in the case when the GT label of an image is unknown.

## 4. New CNN Topology with Parallel Compressed Inputs

**Figure 12:**
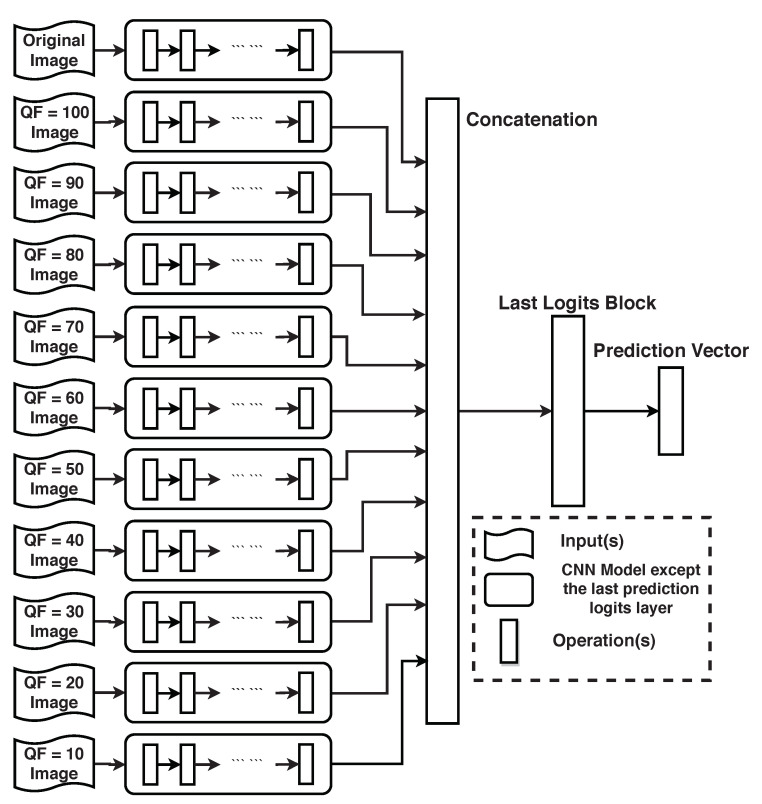
Proposed CNN Topology.

Assume now that the GT label of the original image is unknown. In this case, HRS is not applicable. To utilize compressed versions of the original image to help implicitly the underlying DNN in image classification, in this section, we propose a new CNN topology which is based on the underlying DNN and takes the original input image and its 10 JPEG compressed versions as parallel inputs. This new topology is then trained over the training set of the ILSVRC 2012 dataset and tested on the whole ImageNet validation dataset. We begin with the network architecture of the new CNN topology (shown in Figure 12).

### 4.1. Network Architecture

The underlying DNN is fixed. Consider its main architecture without its last fully connected layer. As depicted in Figure 12, our proposed new CNN topology is based on the underlying DNN and consists of 11 parallel main architectures of the underlying DNN followed by the last fully connected layer at which the logit blocks from the 11 parallel main architectures are concatenated. It takes the original input image and its 10 JPEG compressed versions as 11 parallel inputs, one for each of the 11 parallel main architectures. At its output, we end up with a sorted probability vector of 1000 entries corresponding to the 1000 classes of ImageNet. To alleviate the over-fitting problem [15] and reduce the training complexity, parameters are shared across the 11 parallel main architectures. In other words, the 11 parallel main architectures including their weight parameters inside Figure 12 are identical to each other. This design is also consistent with the architecture in HRS, where the same underlying DNN is used for all selected inputs, regardless of which of the original input image and its 10 JPEG compressed versions is selected.

It is worth pointing out the difference between the above design philosophy and data augmentation. In data augmentation, there is no change in the network architecture itself; however, the underlying DNN is trained over an augmented training set which includes original images as well as their JPEG compressed versions. On the other hand, in the above design, there are fundamental changes in the network architecture; however, the new network topology is still trained over the original training set which contains only original images.

Furthermore, it is important to compare between the proposed network design and Siamese networks. Although both network designs have shared weights in the earlier architecture layers, there are some differences. The purpose of our proposed network design is to improve the classification accuracy of underlying subsequent DNN by inputting the the original input image and its 10 JPEG compressed versions as 11 parallel inputs. The objective function used is the cross entropy loss between the the one hot encoding of the ground truth label and predicted softmaxed logits. Architecture-wise, the proposed topology concatenates the embeddings of 11 parallel inputs to classify the input. However, the purpose of the Siamese network is often comparing different input samples, where the objective function is not to classify input images but to differentiate between them. This objective function is typically defined as the Euclidean distance between the outputs of the sister siamese networks, where embeddings are not concatenated as the proposed topology.

### 4.2. Training Methodology

We trained our proposed topology with stochastic gradient descent (SGD) utilizing the TensorFlow machine learning platform [34]. Our proposed topology used multi-GPU training via two NVIDIA GeForce RTX 2080 Ti GPUs that evenly split a training batch size of 100 for 20 epochs. We utilized the publicly available pre-trained models for Inception V3 and ResNet-50 V2 [32,33] as base models for our proposed CNN topology. For each model, we initialized the learning rate to 0.01 to train the last logit layer with random weights while freezing all its previous modules. When the validation error reaches a plateau with the current learning rate, we stopped the training of the current layer and decayed the learning rate by a factor of 10. Using this reduced learning rate, we incrementally unfroze modules and initialized them with pre-trained weights while using the weights of the previously trained layers. Standard weight decay and batch normalization schemes were applied for Inception V3 and ResNet-50 V2 architectures [9,35]. Inception V3 and ResNet-50 V2 model evaluations were performed using a running average of the parameters computed over time. We decided to halt the training process at the fourth block in Inception V3 and at the second block in ResNet-50 V2 due to limited resources with respect to the amount of memory required by our topology.

### 4.3. Experimental Results

Table 3 shows the Top 1 accuracy and the Top 5 accuracy of our proposed new topology with Inception V3 as its underlying DNN on the whole ImageNet Validation dataset after our proposed new topology was trained for respective layers. In Table 3, “Default” refers to Inception V3 pre-trained with the original images in the training set of the ILSVRC 2012 dataset. The corresponding Top 1 accuracy and Top 5 accuracy results of our proposed new topology with ResNet-50 V2 as its underlying DNN are illustrated in Table 4. Again, in Table 4, “Default” refers to ResNet-50 V2 pre-trained with the original images in the training set of the ILSVRC 2012 dataset. Both tables show that our proposed new CNN topology can consistently improve the Top 1 accuracy of Inception V3 and ResNet-50 V2 by approximately 0.4% and the Top 5 accuracy of Inception V3 and ResNet-50 V2 by 0.32% and 0.2%, respectively.

Although our proposed new topology is structurally more complicated than its underlying DNN, the number of parameters to be trained is essentially the same as that of the underlying DNN due to the fact that the 11 parallel architectures are identical. In Table 5, our partially trained new topology is compared with other state-of-the-art DNNs in terms of both accuracy results and the number of parameters to be trained. In this table, it follows that our partially trained new topology improves the accuracy of Inception V3 and ResNet-50 V2 and provides comparable accuracy performance with respect to other DNNs that have many more parameters to be trained.

In comparison with HRS, the accuracy gain offered by our proposed new topology is smaller, which may increase if the number of trained blocks increases as resources permit. Nonetheless, the results in this section demonstrate that JPEG compression can indeed help improve the accuracy of deep learning even in the case when the GT label of the original image is unknown. Finding better ways to leverage compression to match the classification performance of HRS in the case of unknown GT labels is left open for future research.

## 5. Selectors Maintaining Classification Accuracy While Reducing Input Size

Let us now go back to Figure 2 and continue to assume that the GT label of the original image is unknown to the selector therein. In this section, we present three selectors which maintain the same Top 1 accuracy, the same Top 5 accuracy, and the same Top 1 accuracy and Top 5 accuracy as those of the underlying DNN, respectively, while reducing the size in bits of the input image to the underlying DNN to some degree. These three selectors are referred to as Top 1 Keeper (T1K), Top 5 Keeper (T5K), and Top 1 and Top 5 Keeper (TTK), respectively.

For any original image *I*, T1K selects $I_{j}$ as an input to the underlying DNN if and only if 0≤j≤10 is the largest integer such that the Top 1 label in the sorted $P_{j}$ is the same as that in the sorted $P_{0}$. Similarly, for any original image *I*, T5K selects $I_{j}$ as an input to the underlying DNN if and only if 0≤j≤10 is the largest integer such that the set of Top 5 labels within the sorted $P_{j}$ is the same as that in the sorted $P_{0}$. Likewise, TTK selects $I_{j}$ as an input to the underlying DNN if and only if 0≤j≤10 is the largest integer such that both the Top 1 label in and the set of Top 5 labels within the sorted $P_{j}$ are the same as those in the sorted $P_{0}$, respectively. It is clear that, on any set of images, T1K achieves the same Top 1 accuracy as that of the underlying DNN, T5K achieves the same Top 5 accuracy as that of the underlying DNN, and TTK achieves the same Top 1 accuracy and Top 5 accuracy as those of the underlying DNN.

Table 6 shows the Top 1 accuracy and Top 5 accuracy of T1K and T5K on the whole ImageNet validation dataset when the underlying DNN is Inception V3 and ResNet-50 V2 pre-trained with the original images in the training set of the ILSVRC 2012 dataset, respectively. As seen in this table, T1K degrades the Top 5 accuracy by up to 1.5%, while T5K reduces the Top 1 accuracy by up to 1.26%. However, the advantage is the dramatic reduction in the input size in bits. Table 7 and Table 8 show CR results of T1K, T5K, and TTK for the whole ImageNet validation dataset when the underlying DNN is Inception V3 and ResNet-50 V2, respectively. In Table 7 and Table 8, the default size is the total size in GB of all original images in ImageNet validation dataset, while the new size is the total size of all selected input images by T1K, T5K, or TTK as the case may be. As seen in these tables, the compression ratios achieved by T1K, T5K, and TTK are on average 8.8, 3.3, and 3.1, respectively.

These results demonstrate the advantage of selectors in Figure 2 in terms of input storage savings while roughly maintaining the classification accuracy of the underlying DNN. Applications that require long-term storage of multimedia such as image surveillance will benefit from these selectors.

## 6. Conclusions

In this paper, we formulate a new framework to investigate the impact of JPEG compression on deep learning (DL) in image classification. An underlying deep neural network (DNN) pre-trained with pristine ImageNet images is fixed. For any original image, the framework allows one to select, among many JPEG compressed versions of the original image including possibly the original image itself, a suitable version as an input to the underlying DNN. It was demonstrated that, within the framework, a selector can be designed so that the classification accuracy of the underlying DNN can be improved significantly, while the size in bits of the selected input is, on average, reduced dramatically in comparison with the original image. Therefore, compression, if used in the right manner, helps DL in image classification, which is in contrast to the conventional understanding that JPEG compression generally degrades the classification accuracy of DL.

In the case where the ground truth label of the original image is known to the selector but unknown to the underlying DNN, a selector called Highest Ranking Selector (HRS) is presented and shown to be optimal in the sense of achieving the highest Top *k* accuracy on any set of images for any *k* among all possible selectors. When the selection is made among the original image and its 10 JPEG compressed versions with their quality factor (QF) values ranging from 100 to 10 with a step size of 10, HRS improves, on average, the Top 1 accuracy and Top 5 accuracy of Inception V3 and ResNet-50 on the whole ImageNet validation set by 5.6% and 1.9%, respectively, while reducing the input size in bits dramatically—the compression ratio (CR) between the size of the original images and the size of the selected input images by HRS is 8 for the whole ImageNet validation dataset.

In the case where the ground truth label of the original image is unknown to the selector as well, we also propose a new convolutional neural network (CNN) topology which is based on the underlying DNN and takes the original image and its 10 JPEG compressed versions as 11 parallel inputs. It was demonstrated that the proposed new CNN topology, even when partially trained, can consistently improve the Top 1 accuracy of Inception V3 and ResNet-50 V2 by approximately 0.4% and the Top 5 accuracy of Inception V3 and ResNet-50 V2 by 0.32% and 0.2%, respectively.

Selectors without the knowledge of the ground truth label of the original image are also proposed. They maintain the Top 1 accuracy, Top 5 accuracy, or Top 1 and Top 5 accuracy of the underlying DNN. It was shown that, when applied to Inception V3 and ResNet-50, these selectors achieve CRs of 8.8, 3.3, and 3.1, respectively, for the whole ImageNet validation dataset.

The results in this paper could motivate further developments in at least two directions. First, it would be desirable to develop new compression theory and algorithms for DL to achieve good trade-offs among the compression rate, compression distortion, and classification accuracy, where the compression distortion is for human, and the classification accuracy is for DL machines. Second, the results in this paper imply that the current CNN classifiers are not smart enough and behave as a short-sighted person—if the main features of an object are relatively enhanced and the disturbing features surrounding the object are removed, all through compression, then the CNN classifiers can see the object better. It would be interesting to investigate whether this could be theorized to any Turing classifier (i.e., computable classifier). This first author of the paper believes that this would still be the case, based on insights gained from lossless compression of individual sequences through the lens of Kolmogorov complexity [38].

## Figures and Tables

**Table 1:** Top 1 accuracy and Top 5 accuracy of HRS on the whole ImageNet validation dataset.

Underlying	Default	HRS	Default	HRS
DNN	Top 1	Top 1	Top 5	Top 5
Inception V3	77.6%	83.37%	93.8%	95.79%
ResNet-50 V2	75.58%	80.95%	92.8%	94.64%
Average Diff	-	5.6%	-	1.9%

**Table 2:** Compression performance of HRS for the whole ImageNet validation dataset.

Underlying	Default Size	HRS	CR
DNN	(GB)	Size (GB)	
Inception V3	6.7	0.83	8.1×
ResNet-50 V2	6.7	0.84	8×
Average	6.7	0.84	8×

**Table 3:** Accuracy results of the proposed CNN topology on the whole ImageNet validation dataset when applied to Inception V3.

Layers Trained	Top 1 Accuracy	Top 5 Accuracy
Default	77.6%	93.83%
Logits Block	77.77%	93.880%
From Block10 onward	77.896%	94.094%
From Block9 onward	77.936%	94.11%
From Block8 onward	77.964%	94.15%

**Table 4:** Accuracy results of the proposed CNN topology on the whole ImageNet validation dataset when applied ResNet-50 V2.

Layer Trained	Top 1 Accuracy	Top 5 Accuracy
Default	75.588%	92.828%
LogitsBlock	75.852%	92.982%
From Block4 onward	75.978%	93.012%

**Table 5:** Comparison in terms of the Top 1 accuracy, Top 5 accuracy, and number of parameters to be trained among the state-of-the-art models and our proposed topology. Accuracy results are rounded to the nearest one decimal place. Model name in bold are the models have our topology applied.

Model Name	Top 1	Top 5	# Params
	Accuracy	Accuracy	
VGG 16 [36]	71.5%	89.8%	138M
Inception V1 [7]	69.8%	89.9%	6.8M
Inception V3 [9]	77.6%	93.8%	24M
Inception ResNet-V2 [37]	80.4%	95.3%	56M
ResNet-50 V2 [35]	75.6%	92.8%	25M
ResNet-152 V2 [35]	77.8%	94.1%	60M
ResNet-200 V2 [35]	78.3%	94.2%	65M
**Our topology + Inception V3**	78.0%	94.2%	24M
**Our topology + ResNet 50-V2**	76.0%	93.0%	25M

**Table 6:** Top 1 and Top 5 accuracy results of T1K and T5K on the whole ImageNet validation dataset.

Selector	Inception	Inception	ResNet-50	ResNet-50
	V3 Top 1	V3 Top 5	V2 Top 1	V2 Top 5
Default	77.6%	93.8%	75.58%	92.8%
T1K	77.6%	92.5%	77.58%	91.3%
T5K	76.7%	93.8%	74.32%	92.8%

**Table 7:** Compression ratio results of T1K, T5K, and TTK for the whole ImageNet validation dataset with Inception V3 as the underlying DNN.

Selector	Default Size	New Size	CR
	(GB)	(GB)	
T1K	6.7	0.76	8.8×
T5K	6.7	2.1	3.1×
TTK	6.7	2.3	2.9×

**Table 8:** Compression ratio results of T1K, T5K, and TTK for the whole ImageNet validation dataset with ResNet-50 V2 as the underlying DNN.

Selector	Default Size	New Size	CR
	(GB)	(GB)	
T1K	6.7	0.76	8.8×
T5K	6.7	1.9	3.5×
TTK	6.7	2.0	3.3×

## Data Availability

Data sharing not applicable.
